# Partial Social Integration as a Predictor of COVID-19 Vaccine Rejection and Distress Indicators

**DOI:** 10.3389/fpubh.2022.900070

**Published:** 2022-07-26

**Authors:** Yohanan Eshel, Shaul Kimhi, Hadas Marciano, Bruria Adini

**Affiliations:** ^1^Stress, and Resilience Research Center, Tel Hai College, University of Haifa, Haifa, Israel; ^2^ResWell—Multinational Resilience and Well-Being Research Collaboration, Tel Aviv University, Tel Aviv, Israel; ^3^The Institute of Information Processing and Decision Making (IIPDM), University of Haifa, Haifa, Israel; ^4^Department of Emergency and Disaster Management, ResWell—Multinational Resilience and Well-Being Research Collaboration, School of Public Health, Sackler Faculty of Medicine, Tel Aviv University, Tel Aviv, Israel

**Keywords:** partial social integration, distress symptoms, sense of danger, societal resilience, vaccine rejection

## Abstract

Partial social integration refers to the perceived exclusion of individuals or groups, from full participation in their society. The current study claims that perceived partial social integration (PPSI) constitutes a substantial predictor of the rejection of the COVID-19 vaccine, a significant mediator of the impact of demographic variables (such as age and level of income) on this vaccine rejection, and an important predictor of indices of psychological distress during pandemic times. Previous publications show that although vaccines constitute a very efficient means for countering pandemics, vaccine hesitancy is a prevalent public response to the COVID-19 pandemic. The present study is one of a few studies examining the impact of psychological variables on the actual behavior of vaccine rejection rather than on the cognitive element of vaccine hesitancy. A sample of 600 Israeli Jewish adults responded in February 2022 to an anonymous questionnaire exploring, among other issues, the (PPSI), the individual level of vaccine uptake, and the level of distress of these individuals. Path analyses of the variables predicted by PPSI indicated the following results: (a) PPSI score negatively predicted vaccine uptake level and significantly mediated the effects of age and family income on the level of vaccination. (b) PPSI levels significantly predicted higher levels of anxiety, depression, and a sense of danger and negatively predicted societal resilience. The discussion elaborates the contention that the PPSI is a substantial cause of psychological distress and in compliance with the pandemic vaccination guidelines, despite the potential health risk involved.

## Introduction

Social integration which includes cohesion, group identification, and social support, can be defined as the extent to which individuals participate in a variety of social relationships, and regard themselves as a basic part of their social unit (1, 2). A sense of integration in the community has been associated with feelings of competence and control, and it constitutes an important component of psychological wellbeing (3–5). Perceived partial social integration (PPSI) has been defined as a way through which individuals or groups are wholly or partly excluded from full participation in the society in which they live (6). Individuals who feel more strongly that they are only partly integrated (or partly excluded socially), may be characterized, for instance, by a lower subjective social or economic standing, greater perceived discrimination and low perceived control of one's life, experience higher levels of psychological stress (7). This higher levels of distress may result in inhibition from initiating or maintaining social contact with others (8, 9), so this may be a kind of vicious circle that affects the psychological and societal condition of these people. Individuals who engage in social interactions generally expect inclusion (10, 11). This expectation is based on the individuals' need to establish and maintain social connections in the service of psychological wellbeing (12). Rather than inclusion, PPSI involves an experience of social exclusion, which makes people feel interpersonal rejection and social discrimination (https://www.frontiersin.org/articles/10.3389/fpsyg.2017.00112/full - B47) (13). We claim that perceived, rather than actual exclusion, and even perceived partial integration into a desired social section, are sufficient reasons for feeling distressed. The current paper explores this argument with data relevant to vaccine uptake during the COVID-19 pandemic in Israel.

### Partial Social Integration (PPSI) and the COVID-19 Vaccine Rejection

The COVID-19 pandemic has infected, to date, millions of people and caused wide mortality worldwide (14). The available data show that vaccines constitute the most successful public health intervention, for containing infectious diseases (15). However, a substantial number of people worldwide, reveal vaccine hesitancy despite this serious health threat. A scoping review of COVID-19 studies across four continents (16), reports that approximately half of the available studies indicate vaccine hesitancy rates of 30% and more. Furthermore, a recent longitudinal study conducted in the USA, shows that the increased salience of a disease threat has been accompanied by a decline in the general intentions of getting the pandemic vaccine (17). Israel is among the countries with the highest levels of vaccination for COVID-19, with 78% of those 12 years or older fully vaccinated (18). Nevertheless, several studies indicate that the Israeli public is not free of COVID-19 vaccine hesitancy [e.g., (19, 20)].

A large number of studies investigate vaccine hesitancy by measures of readiness and unwillingness to accept the COVID-19 vaccine [e.g., (21, 22)]. Aw et al. (16), show that COVID-19 vaccine hesitancy is more prevalent among specific social sub-groups: females, younger adults, having a non-White ethnicity, and having a lower education or income levels. It has been found further, that vaccine uptake was lower amongst some ultra-orthodox Jewish parties, as well as low socio-economic status communities (23). Peretti-Watel et al. (24) claim that vaccine hesitancy is an ambiguous notion and its theoretical background appears uncertain. Their theoretical analysis defines this phenomenon as “a kind of decision-making process that depends on people's level of commitment to healthism/risk culture and their level of confidence toward health authorities and mainstream medicine.” Rather than examining the origin of the process of vaccine hesitancy or the attitudes of the respondents toward vaccination, the present study employs a behavioral measure: the number of vaccine uptakes made by each of them.

Several studies claim that COVID-19 vaccine hesitancy and rejection are characteristic of individuals and groups experiencing anxiety and distress (25, 26) but they fail to provide a sufficient explanation connecting belonging to specific social groups with COVID-19 vaccine hesitancy or rejection. We claim that a high level of stress does not constitute a sufficient reason for vaccine rejection. Research shows, for example, that compared to men, women reported higher levels of anxiety and fear, as well as greater life disturbance during the COVID-19 pandemic (27). However, Israeli women do not reject vaccination to a greater extent than men do (28). We posit, therefore, that this vaccine rejection is not determined by the anxiety and distress *per se*, but rather by regarding oneself as only partly socially integrated, namely, partly socially excluded. We assume that a higher level of PPSI will result in vaccine rejection, and will characterize several populaces that regard themselves as partly socially excluded. This vaccine rejection will be enhanced by the following demographic variables: being young adults (28), possessing lower education and/or lower-income, as well as by being ultra-orthodox religious. We assume that this stressful experience of feeling perceived partial social integration will positively predict levels of individual distress and perceived danger, and will negatively predict trust in public authorities and societal resilience. Furthermore, we assume PPSI level will positively predict vaccine rejection and due to the nature of individuals belonging to these demographic groups, will mediate their effects on this rejection. It is interesting to note that the PPSI notion is somewhat similar to (29) claim that vaccine hesitancy is sometimes mediated by experiences of social exclusion. These experiences impair citizen-government trust and undermine a climate of social connectedness. Furthermore, these experiences lead many marginalized individuals to resist vaccination as a form of agency or to avoid vaccination. The association of the following variables with PPSI scores and with vaccine rejection was examined in the present study.

### Societal (Formerly Referred to as National) Resilience

This concept refers to trust in the ability of one's state and its leadership, to successfully deal with adversity or threats, and to recover as quickly as possible after the threat has been removed (30, 31). Societal resilience has negatively predicted stress symptoms and has positively predicted posttraumatic recovery across three age groups (32). We assume that level of societal resilience will be predicted by higher PPSI scores since feeling oneself a kind of social outsider is likely to be associated with low trust in social leaders.

### Distress Symptoms

Distress symptoms are the most common negative human reactions in response to threats and or disasters. Among the common reactions are symptoms of anxiety and depression (33). Several researchers use the level of the individual's stress symptoms as a measure of the individual's resilience and/or coping level (34). It has been found that belonging to the socially excluded groups mentioned above, who regard themselves as only partly socially integrated, is associated with a higher level of distress as well as a higher vaccine rejection (28).

### Sense of Danger

Threats and disasters often evoke feelings of danger, mainly the individual's feelings that his/her life and/or family life are in danger (35). These feelings, like symptoms of stress, are negative indicators of an individual's coping (36) which are expected to associate positively with PPSI levels.

### Young Adults

Developmental psychologists believe that human development is carried out by consecutive stages, one of which is young adulthood (37, 38). Young adults are faced with the need of relying to a greater extent on their resources, in less structured and familiar circumstances. The person-context interactions during young adulthood are many and complex, leading to multiple potential pathways. Young adults have no way to know in advance whether they will embark on a positive trajectory, or will they experience a negative trajectory in the spheres of education, vocation, relationships, and health status (39). Young adults, who are well aware that their participation in the grownup society is not completed yet, are likely to wonder what their lives will look like in the future, how they will find a mate and raise a family, and whether they will succeed in establishing a desired social or professional position in the future (40). There is no clear definition for the developmental stage of young adulthood, but since its developmental tasks are attained at different stages, it has been argued that the consolidation of adult status is likely to be achieved closer to the end of the third decade of life (41). Rather than addressing a specific age as “young adulthood” we analyze in the present study age as a continuous variable and claim that the younger the age the higher the PPSI score and the lower the level of vaccination. The younger age of adults has been found as the best predictor of vaccine rejection (28).

### Socioeconomic Status

It has been found that the likelihood of the COVID-19 vaccine rejection and hesitancy in Saskatchewan, Canada is increased by lower education levels as well as by lower financial conditions (42), even when the vaccination is free of charge (28). The empirical research indicates further that social class, characterized mainly by levels of income and education, affects thoughts, feelings, and behavior (28). For example, there is growing evidence that income inequality is associated with mental health outcomes and may cause anxiety, clinical depression as well as a low self-perception (43). Manstead (44) suggests that the cycle of disadvantage starts with poor material conditions and ends with lower opportunities for entering and succeeding socially and economically, as well as low social mobility. There is solid evidence that the material circumstances in which people develop and live their lives have a profound influence on how they construe themselves and their social environments [e.g., (45, 46)].

The resulting differences in the thinking and acting of lower-class people in contrast to higher-class people reinforce these influences of social class background, making it harder for working-class individuals to achieve mobility and change their social position. Lower-income and lower education levels are two attributes that are likely to make people feel that their chances of improving their living conditions are rather scarce since they are already partly excluded by the general society (47). Therefore, we expect that lower-income and educational levels will constitute two additional predictors of PPSI, which will be associated as well with a lower degree of vaccination.

### Orthodox Religiosity

A review of the research confirms that extremely religious people are more prone to vaccine hesitancy (16). Ultra-orthodox Jews frequently wish to exclude themselves from the secular way of life of the general Israeli society and live as a separate social entity in closed communities (48). Ultra-orthodox Jewish communities trust their religious leaders, who have little confidence in the motives of the secular authorities, rather than the general health system in keeping the COVID-19 precaution measures (49). A recent COVID-19 study (28) indicates that more devoted religious Jews in Israel are vaccinated to a lesser degree than the general population. Four categories determine self-definitions of the level of religiosity of Jewish people in the Israeli context. The first category, secular, is held by most Israeli Jews, who do not regard themselves as religious. The second category, traditional, characterizes individuals who keep some of the commandments of the Jewish religion and some of its traditional habits. The third category, religious, is held by people who are committed religious believers who perform all the religious commandments. The fourth, ultra-orthodox category includes those who adhere to a very strict religious way of life, devote their time to learning the Holy Scriptures, and generally refrain from acquiring any scientific or general education.

It should be noted that religious reasons affecting vaccine hesitancy and rejection characterize several religious groups, including Protestants, Catholics Jewish, Muslims, Christians, Amish, Hinduist, and Sikhist (50). Muslims in north Pakistan rejected the polio vaccine for religious reasons, due to a belief that the will of God, rather than vaccination, determines health and sickness (51). Followers of Hinduism and Sikhism rejected the polio vaccine believing that it opposed some of their religious taboos (52). Ethical objections to vaccines including fetal cells were raised in Amish communities (53) and by senior catholic leaders from the US and Canada (54).

To sum, according to the above discussion concerning the associations of PPSI with different variables and indicators, the following three hypotheses are investigated:

- The level of PPSI will positively predict symptoms of distress and the level of sense of danger, and will negatively predict societal resilience.
- PPSI will positively predict the level of vaccine rejection and will mediate the prediction of this rejection by the demographic characteristics of age, levels of education and income, and level of religiosity.
- In line with previous studies, women will report higher levels of distress compared to men. However, since their mean PPSI score will not differ significantly from the men's score, they will not differ from men on the level of vaccination.

## Methods

### Data Collection and Analysis

The data were collected via an internet panel company possessing a database of above 65,000 residents from all demographic sectors and geographic locations of Israel (https://sekernet.co.il/) (accessed along four days on the first half of March 2022). Eligible to participate in the study were adults >18 years old. A stratified sampling method was employed, aligned with the data of the Israeli Central Bureau of Statistics (55), appropriately representing the varied groups of the Israeli Jews population (regarding gender, age, and geographic dispersal). The questionnaire was approved by the Ethics Committee of Tel Aviv University (Study no. 0001150-2) and all the participants signed an informed consent form. The distribution of the questionnaires was stopped once the agreed number of participants was reached. We used path analyses to test the hypothesis that the level of PPSI will positively predict symptoms of distress and the level of sense of danger, and will negatively predict societal resilience, as well as the hypothesis that PPSI will positively predict the level of vaccine rejection and will mediate the prediction of this rejection by the demographic characteristics of age, levels of education and income and level of religiosity. It is important to note that in a saturated model, there is no need to examine a model fit as the default and the saturated model are the same (56). The gender differences in PPSI, level of distress, and vaccine rejection were examined by analysis of variance (ANOVA).

### Participants

The present sample includes 600 Jewish adults representing all components of the Israeli Jewish population. Table 1 presenting their demographic variables shows that their ages range from 18 to 84 years, 51% of them are females and 49% are males. They represent wide ranges of religiosity, income, and education levels, as well as a wide range of political attitudes. 78.2% of them reported that they have been vaccinated three or four times.

**Table 1:** Demographic characteristics of the participants.

**Variable**	**Category**	***N***	**Percent**
Age groups	18–30	178	29
	31–40	124	21
	41–50	124	21
	51–60	80	14
	61–82	90	15
Religiosity	Secular	257	43
	Traditional	215	36
	Religious	67	11
	Orthodox	61	10
Gender	Male	291	49
	Female	309	51
Family income compared to average	1. Much lower	167	29
	2. Lower	115	20
	3. Average	119	21
	4. Higher	77	14
	5. Much higher	32	6
	No response	55	10
Education	1. Elementary	8	1.3
	2. Partial high school	60	10
	3. High school	138	23
	4. Partial academic	134	22
	5. Bachelor's degree	181	30
	6. Master's degree +	79	13

### Measures

#### Level of Vaccine Uptake

Israeli residents are requested, to date (February 2022), to be vaccinated at least three times against COVID-19. Specific vulnerable populations were called to be vaccinated with an additional (fourth) booster vaccine. The degree of vaccine uptake was determined by a single item: “To what extent are you currently vaccinated against the COVID-19?” The five-point response scale ranges from 1 = not vaccinated, to 5 = vaccinated four times. It is important to note that the present study examines reports of actual vaccination behavior rather than vaccine hesitancy that tends to measure vaccine willingness or an attitude concerning vaccine uptake [e.g., (21)].

#### Perceived Partial Social Integration

The PPSI in the context of the COVID-19 vaccination was determined by a six-item scale about the COVID-19 pandemic, which has been designed for the present study. We believe that the major issues that trouble individuals who regard themselves as only partly socially integrated include questions of one's social status, retaining free will under conditions of inequality, and being unappreciated and unaccepted by others (see Table 2). The PPSI scale pertains to regarding oneself as only partly integrated into one's society. The response scale ranged between 1= not true at all, and 5 = very much true. The reliability of this scale in the present sample was good (Cronbach's α = 0.79).

**Table 2:** Perceived partial social integration (PPSI) (COVID-19) scale.

**Concerning vaccinations against COVID-19, to what extent do you perceive each of the following items to be true regarding yourself?**	**Not at all**	**To a small extent**	**To a moderate extent**	**To a great extent**	**To a very great extent**
1. Only I am responsible for my health and no one is authorized to tell me whether to be vaccinated or not	1	2	3	4	5
2. I do not receive equal status as the rest of the people in my country	1	2	3	4	5
3. In the current situation, I do not feel that the government's regulations concerning vaccinations are binding on me	1	2	3	4	5
4. It's my right to be treated as I deserve before I am required to get vaccinated	1	2	3	4	5
5. The pressure that is put on me to get vaccinated infringes on my freedom	1	2	3	4	5
6. The state does not help me when I need assistance, so I did not fulfill its demand to get vaccinated	1	2	3	4	5

#### Distress Symptoms

The BSI scale was employed (57). The present study included the four items about anxiety (example: Feeling tensed or keyed up) and the five items on the depression sub-scale (example: Feeling hopeless about the future). The response scale to this questionnaire ranges from 1 = not at all to 5 = to a very large extent. Respondents were asked to report the extent to which they are currently suffering from any of the problems presented. The internal reliability of the anxiety scale was good (Cronbach's α = 0.72), and the reliability of the depression scale was high (α = 0.87).

#### Sense of Danger

Sense of danger scale includes six items (58). We have used a shortened version of this scale due to its good reliability. No new items were added. The original items of sense of danger were employed and were associated with the COVID-19 pandemic. For example: “To what extent do you feel your life is in danger due to the coronavirus?”; “To what extent do you feel that the lives of your family members or those who are dear to you are in danger due to the coronavirus?” The response scale of the sense of danger index ranges from 1 = not at all, to 5 = to a very large extent. Good reliability was found for this scale in the present study (Cronbach's α = 0.81).

#### Societal (National) Resilience

The original scale (31) is based on four main social components that have been attributed in previous studies to societal resilience: patriotism, optimism, social integration, and trust in political and public institutions. This index has received much research support, both in Israel (59) and in other countries (30). The original scale employed in previous resilience studies during the COVID-19 pandemic included 16 items. In the current study, we have used an abbreviated version that included 5 items. Example item: “I have full confidence that the Israeli government makes the appropriate decisions in managing the COVID-19 crisis”. The response scale for the societal resilience items ranges from 1 = strongly disagree to 6 = strongly agree. The reliability of the scale in the current study was high (Cronbach's α = 0.94).

#### Young Adulthood

Respondents indicated their age in years.

*Religiosity* was determined by the question “How would you define your level of religiosity?”. The four response options were: (1) Secular, (2) Traditional, (3) Religious, (4) Ultra-orthodox.

*The family income level* was established by the following item: ”The average income of an Israeli family today is 18,671 NIS per month. Your family's income is (1) Much lower than this average; (2) Lower than this average; (3) Around this average; (4) Higher than this average; (5) Much higher than this average.

*Education level* was determined by the item “What is your education level?” The six response options were: (1) Primary education, (2) Partial secondary education, (3) Secondary education, (4) Partial academic education, (5) Bachelor's degree, and (6) Master's degree or higher.

## Results

**Figure 1:**
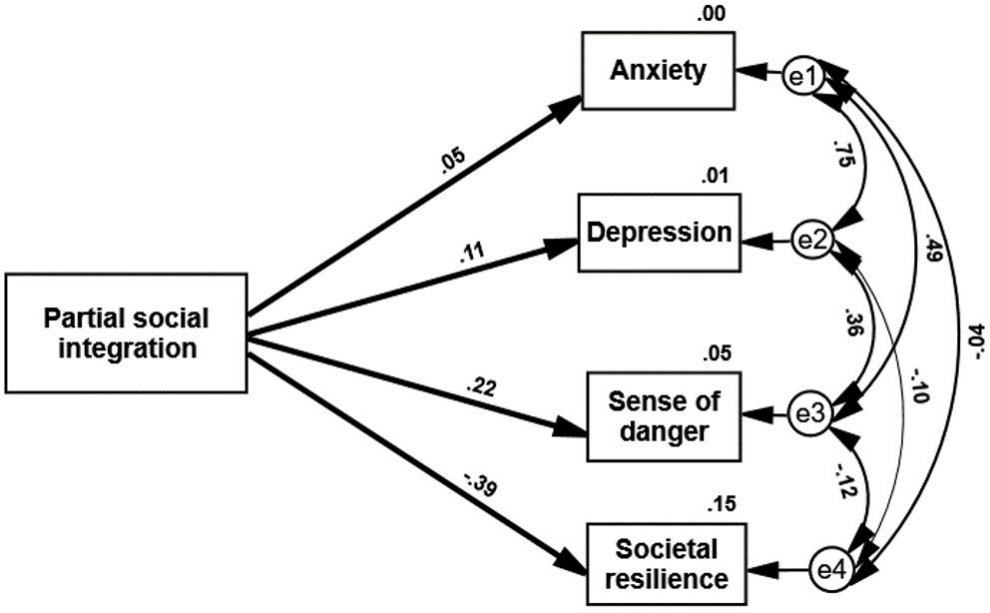
Path analysis—PPSI score predicting measures of distress and societal resilience. All paths are significant (*p* < 0.05).

Hypothesis (a) claimed that the PPSI score will positively predict distress symptoms as well as the sense of danger, and will negatively predict societal resilience: The higher partial social integration, the higher distress, and lower societal resilience and vice versa. A path analysis that was conducted fully supported this hypothesis (see Figure 1). These results indicate that perceived partial social integration indeed results in a high level of distress, and a distrust in the state's authorities.

**Figure 2:**
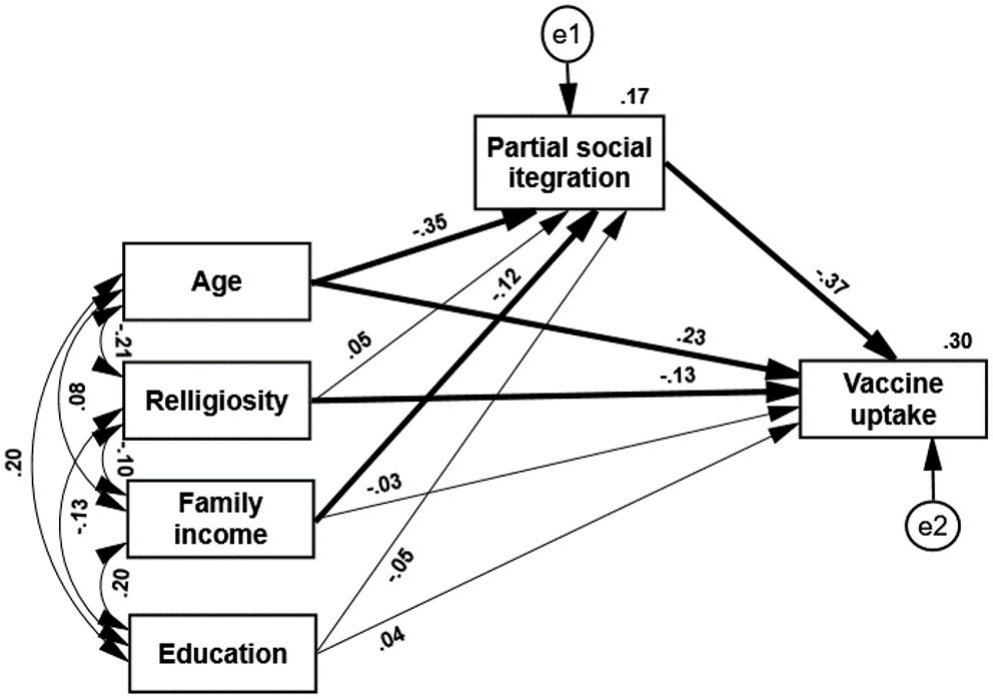
Path analysis—PPSI mediates the prediction of vaccine uptake by four demographic variables. Paths marked with a thick line symbolize a significant path (*p* < 0.05).

The second hypothesis stated that PPSI will positively predict vaccine rejection and will mediate the impact of demographic characteristics (age, level of religiosity, family income, and education) on vaccine rejection. Second path analysis indicated that PPSI was indeed a substantial predictor of vaccine rejection, which mediated the prediction of age and fully mediated the prediction of family income and vaccine rejection. PPSI did not mediate the association between education or religiosity and vaccine rejection (see Figure 2). The results show that demographic variables (in the present case, age and family income) significantly predict the level of vaccination. More importantly this prediction of vaccination by two of the demographic variables—age and income—is significantly mediated, as hypothesized, by the level of PPSI. Additionally, this path analysis indicated the following: The five predictors explained 30% of vaccine uptake variance; age was the best predictor of partial social integration. This path analysis shows that PPSI scores are significantly and negatively affected by the age of the respondents and by their family income. Older and more affluent people regard themselves as more integrated into their society. Vaccine uptake is positively predicted by age and negatively predicted by religious orthodoxy. Younger people and more religiously orthodox individuals refrain more often from being vaccinated. These results have mainly supported our second hypothesis.

Hypothesis (c) claimed that though women in the Israeli context will report higher levels of distress compared to men, no significant gender difference will be found in their mean PPSI score or level of vaccination. An analysis of variance comparing these variables of males and females supports this hypothesis. As can be seen in Table 3 women indeed reported higher levels of distress compared to men (*p* < 0.05). However, they did not score higher than men on the PPSI scale, and their level of vaccination did not differ significantly from the men's level of vaccine uptake. In other words, gender does not predict the level of vaccination in this Israeli sample, without the mediation of PPSI. This finding fully supports our third hypothesis.

**Table 3:** Univariate analyses of variance comparing males and females, means, standard deviations, and f values (*n* = 600).

**Variable**	**Male (*****n*** **=** **291)**		**Female (*****n*** **=** **309)**		
	**Mean**	**SD**	**Mean**	**SD**	**F**
Anxiety	2.29	0.83	2.44	0.89	4.14^*^
Depression	2.01	0.85	2.16	0.96	4.26^*^
Sense of danger	2.00	0.86	2.21	0.94	8.15^**^
Partial integration	3.24	0.94	3.22	0.86	0.46
Vaccine uptake	2.78	0.84	2.73	0.77	0.62

* *p < 0.05*,

** *p < 0.01*.

## Discussion

The present study substantiates the significant role of perceived partial social integration (PPSI) in determining the well-being of individuals and in directing their behavior in the context of the COVID-19 pandemic. Studies of social desegregation emphasize the negative consequences of actually keeping social groups apart, based on their demographic characteristics. For example, it has been shown that living in a highly segregated inner-city neighborhood often limits black and minority residents' chances of escaping poverty, deprivation, and isolation due to poor social networks, limited local resources and constrained job opportunities (60–62). Previous research shows that a non-threatening psychological climate, characterized by comradeship and mutual support, is encouraged by open and fluid communication, whereas undesirable life events negatively influence social integration and participation over time (63). Psychological research indicates, therefore, that segregation is associated with negative emotional outcomes (64).

The present study demonstrates that perceptions of being only partially integrated into the desired society promote high levels of distress and result in low levels of societal resilience. Our data support the contention that PPSI consistently decreases psychological coping, and is experienced as a continuous stressful and depressing condition. Such personal feelings of stress due to perceived inequality and low social appreciation result, as expected, in mistrusting the authorities, and eventually in the case of COVID-19, also in vaccine rejection.

Social research tends to regard integration and segregation as a dichotomy and inclines to ignore the intermediate range between these two ends. The present new PPSI scale reveals the more covert aspects of social acceptance and rejection. It emphasizes the major psychological role of a rather common stressful condition, in which individuals regard themselves as neither being segregated nor being integrated members of their society, since they feel only partially integrated. The present results clearly show that the emotional effects of such PPSI, which do not amount to actual social exclusion, impacts substantially people's perspective on life and their behavior.

Faced with the request to be vaccinated against the COVID-19 virus while the pandemic is spreading, those who regard themselves as only partly socially integrated or as partly socially excluded, distrust the intentions and the goodwill of the authorities (65). Betsch et al. (66) claim that the five main individual-level determinants of vaccine hesitancy: are confidence, complacency, convenience (or constraints), risk calculation, and collective responsibility. The present results indicate that actual vaccine rejection, rather than deliberating about vaccination, is predicted by PPSI scores, that is by one's perceived social standing.

Many of those who regard themselves as only partly integrated prefer to express their frustration and antagonism, by refraining from taking the vaccine doses recommended by the public health system. In the name of freedom of choice and human rights, they ignore the authorities' request for vaccination, at the expense of their health interest. This behavior is carried out despite available data, covering the period from January to October last year in England, indicating that the rate of death from COVID-19 was 96% lower in people who had received a second dose of vaccine than in those who were unvaccinated (67).

It is interesting to note that as indicated in Figure 1 age was the best demographic predictor of vaccine rejection. The levels of education, religiosity, and socio-economic status do not change easily throughout life, whereas the world of young adults is much more dynamic and presents them quite constantly with new dilemmas. Young adults have to cope successfully with new and changing challenges despite their inexperience in performing them. Improving one's working skills and social standing are never-ending targets, much the same as developing positive and steady marital relations, or raising a family. Their future depends on fruitfully overcoming a host of obstacles without any guarantee of success. They do their best to belong to the adult world and are aware of the fact that this is their time to develop and succeed, but while comparing themselves to other young adults they keep wondering how successful they are in achieving their goals.

Our finding that more orthodox religion negatively impacts the level of vaccine uptake is supported by other studies. Frei-Landau (49) as well as Zalcberg and Block (68) report that some ultra-orthodox Jewish communities tend to trust their religious leaders more than the general health system in keeping the COVID-19 precaution measures. The significant effect of religion on health behavior in general and on the COVID-19 vaccine uptake is not limited to the ultra-orthodox Jews investigated in the present study. It is characteristic of different religions in different parts of the world due to devotion to religious commands or highly regarded religious leaders (50).

Figure 2 (69) indicates further that the PPSI score does not mediate the association of religiosity with the level of vaccine uptake. Two reasons can explain this finding. First, the level of vaccination of ultra-religious Jews is strongly affected more often by the decrees of their religious leaders than by scientific facts presented by the public health system (68). Second, their relatively low sense of perceived integration reflects a wish to separate themselves from the general society, and a reluctance to be a part of a secular state, rather than a disappointment of living at the margin of this society. The PPSI score did not mediate as well the link between the level of education and vaccine uptake. This result may reflect the fact that the investigated sample is biased toward higher education. 65% of the participants hold semi-academic or academic degrees. These individuals are likely to feel more integrated into the general society and are less prone to be affected by conspiracy theories about the COVID-19 vaccine.

## Limitations

The concept of partial social integration and its behavioral outcomes have not been studied so far in depth. Further studies that will be conducted under different stressful conditions and in varied cultural settings would be required to support the concept of PPSI and its social consequences, the PPSI scale, and the present findings. Furthermore, the present correlative study is not enough for determining causality. Different research methods are required to establish the claim that the experience of PPSI is responsible for the reported psychological and behavioral findings. An additional potential limitation concerns the present sampling process. The sample was drawn from a large database but there is no indication of response rates or differences between those who responded and those who did not. There is no way to determine the extent to which the present sample constitutes a representative sample of the Israeli population.

## Conclusions

The present study investigated the role of perceived partial social integration (PPSI) in determining the wellbeing of individuals and in directing their actual behavior during the COVID-19 pandemic. Our results indicate that PPSI is a powerful psychological determinant of individual level of distress which constitutes concurrently an important predictor of vaccine rejection. PPSI provides a theoretical explanation for the findings that people who are not feeling secure about their social belonging (such as young adults or economically disadvantaged people) are rejecting vaccination more often than the general public. It shows that a common feature of these groups is their perception of being only partly socially included, which is shared by vaccine rejecting individuals (70). Furthermore, PPSI mediates the associations of some of these groups with vaccine rejection.

## Data Availability Statement

The raw data supporting the conclusions of this article will be made available by the authors, without undue reservation.

## Ethics Statement

The studies involving human participants were reviewed and approved by Tel Aviv University Ethics Committee, Tel Aviv University. The patients/participants provided their written informed consent to participate in this study.

## Author Contributions

YE conceptualized and initiated the study. SK and BA designed the methodology including the data collection. YE and SK analyzed the data. YE drafted the first draft. All authors reviewed the manuscript. All authors contributed to the article and approved the submitted version.

## Conflict of Interest

The authors declare that the research was conducted in the absence of any commercial or financial relationships that could be construed as a potential conflict of interest.

## Publisher's Note

All claims expressed in this article are solely those of the authors and do not necessarily represent those of their affiliated organizations, or those of the publisher, the editors and the reviewers. Any product that may be evaluated in this article, or claim that may be made by its manufacturer, is not guaranteed or endorsed by the publisher.
